# The brittle-ductile transition in active volcanoes

**DOI:** 10.1038/s41598-018-36505-x

**Published:** 2019-01-15

**Authors:** Francesco Parisio, Sergio Vinciguerra, Olaf Kolditz, Thomas Nagel

**Affiliations:** 1 Department of Environmental Informatics, Helmholtz Centre for Environmental Research GmbH – UFZ, Leipzig, Germany; 2 Dipartimento di Scienze della Terra, Universitá di Torino, Turin, Italy; 3 Applied Environmental Systems Analysis, Technische Universität Dresden, Dresden, Germany; 4 Chair of Soil Mechanics and Foundation Engineering, Institute of Geotechnics, Technische Universität Bergakademie Freiberg, Freiberg, Germany

## Abstract

Contrasting deformation mechanisms precede volcanic eruptions and control precursory signals. Density increase and high uplifts consistent with magma intrusion and pressurization are in contrast with dilatant responses and reduced surface uplifts observed before eruptions. We investigate the impact that the rheology of rocks constituting the volcanic edifice has on the deformation mechanisms preceding eruptions. We propose a model for the pressure and temperature dependent brittle-ductile transition through which we build a strength profile of the shallow crust in two idealized volcanic settings (igneous and sedimentary basement). We have performed finite element analyses in coupled thermo-hydro-mechanical conditions to investigate the influence of static diking on the local brittle-ductile transition. Our results show that in active volcanoes: (i) dilatancy is an appropriate indicator for the brittle-ductile transition; (ii) the predicted depth of the brittle-ductile transition agrees with the observed attenuated seismicity; (iii) seismicity associated with diking is likely to be affected by ductile deformation mode caused by the local temperature increase; (iv) if failure occurs within the edifice, it is likely to be brittle-dilatant with strength and stiffness reduction that blocks stress transfers within the volcanic edifice, ultimately damping surface uplifts.

## Introduction

The classical interpretation of pre-eruptive patterns proposes that magma intrudes within the basements of volcanic edifices from the magma chambers, ascending toward the surface throughout repeated episodes and feeding eruptive cycles. This can generate high seismic activity, pressurisation/depressurisation, deformation of the volcanic edifice, inflation and mass redistributions^1–3^. Other observations have shown limited or contrasting mechanisms before eruption occurrence: dilatant responses to changes in the stress field^4^, ambient seismic noise^5^ and inverse density (gravity) changes with limited or localized uplifts and seismicity^6,7^. The mechanical properties play a key role on magma transport from deep storage zones to the surface^8^ and the local stress variations induced by propagating dikes^9^ at shallower levels control the temperature changes and the occurrence and timing of the eruption^2^. Observation of limited surface uplifts and inverse density changes within the edifice have challenged this interpretation^7^. We propose a new departure in which the rheology of the rocks influences pre-eruptive processes by constraining the brittle-ductile transition (BDT) depth within volcanic basement.

Deformation and failure mode transition between brittle-cataclastic-localized patterns into ductile non-localized plastic-flow occurs at increasing temperature and mean stress in the earth’s crust^10–12^. However, while the average thermal gradient worldwide is around 30 K km^−1^, in volcanic areas it can exceed 150 K km^−1^, modifying the mechanical properties of rocks at much shallower depth, hence uplifting significantly the BDT. Because mechanical properties are strongly influenced by lithology, BDT depth is normally different for sedimentary or igneous volcanic basements. Increase in rock’s temperature and pressure of magmatic fluids induced by intruding dikes is also expected to control (locally) the BDT and, as a consequence, the earthquakes distribution associated with diking^13^. Failure in brittle regime exhibits dilatancy, followed by fracture nucleation, growth and propagation with the mechanical properties of strength and stiffness degrading rapidly with load increments. Hence, it is likely that volumes in the edifice located above the BDT fail in brittle regime, causing dilatancy and stiffness degradation. If this is the case, such decrease of density is at the opposite of the intuitive increase expected to happen during magma intrusion. We believe that the BDT, which is controlled mainly by temperature, pore pressure, stress and lithology, plays a fundamental role in controlling several pre-eruptive processes.

To prove this hypothesis, we have developed a constitutive model based on experimental results on two rocks that can be considered—from the point of view of the mechanical properties—as end terms of lithologies forming strato-volcanic edifices: a carbonate (Comiso Limestone^14^) and a basalt (Escandorgue Basalt^15^). Although the literature on models of thermo-plastic failure^16–18^ and brittle-ductile transition^19–22^ of earth materials is relatively abundant, specific models encompassing both mechanisms of temperature and pressure dependence for strato-volcanoes, avoiding over-parametrisation, are still missing. The primary goal of this study is to assess deformation mode at failure onset. Hence, a limit surface (yield function) in the stress-temperature space provides the onset of inelastic deformations and represents the strength envelope of the material.

The experimental data on which the model is based are obtained from published results of triaxial tests at high temperature and high pressure^14,15^ (see Methods for additional details). Experimental results of Comiso limestone have shown brittle deformation up to 300 °C, and up to 200 °C, at confinements of 50 MPa and 100 MPa respectively^14^. Weakening at higher temperature is promoted by enhanced solid state diffusion mechanisms and intra-crystalline plasticity^14^, though mass loss via calcite and dolomite dissociation reactions, which are accelerated by temperature increase, cannot be excluded^23^. For basalt, the authors reported that the samples at 100 MPa confinement always localized and the ones at 300 MPa confinement localized for 600 ≤ *T* ≤ 800 °C^15^. Thermal weakening could be caused by both diffuse micro-cracking and plastic deformation of the minerals; intra-crystalline plasticity was rarely observed^15,24^.

Because dike intrusions, wether propagating sub-horizontally from a central conduit^25^ or from eccentric reservoirs bypassing it, are widespread within the volcanoes and can cause local heat and pressure increases, we have investigated their effects on the rheology of the host rocks. This process can potentially expose large volumes of fresh and unaltered rocks to high temperature, influencing its deformation mode (brittle or ductile)^23^. Although the relationship between dike intrusion and seismic swarms has been widely studied in volcano seismology^1,26–37^, a clear link between seismic migration and eruption onset is not yet established^38^. Despite dikes are relatively small bodies and bias can be related to the hypocenter locations due to network coverage^37^, debate still exists on whether seismic swarms are driven by the propagation of the dike tip itself^39,40^ or are instead the result of the stress changes induced by the intrusion processes and do not necessarily track the dike tip during propagation^41^. Here we study the relationship between diking processes and rheology by investigating the effect that a heat and pressure source (dike intrusion) has upon the BDT location within the host rock. To do so, we have performed coupled thermo-hydro-mechanical finite element (FE) simulations.

## Results and Discussion

**Figure 1:**
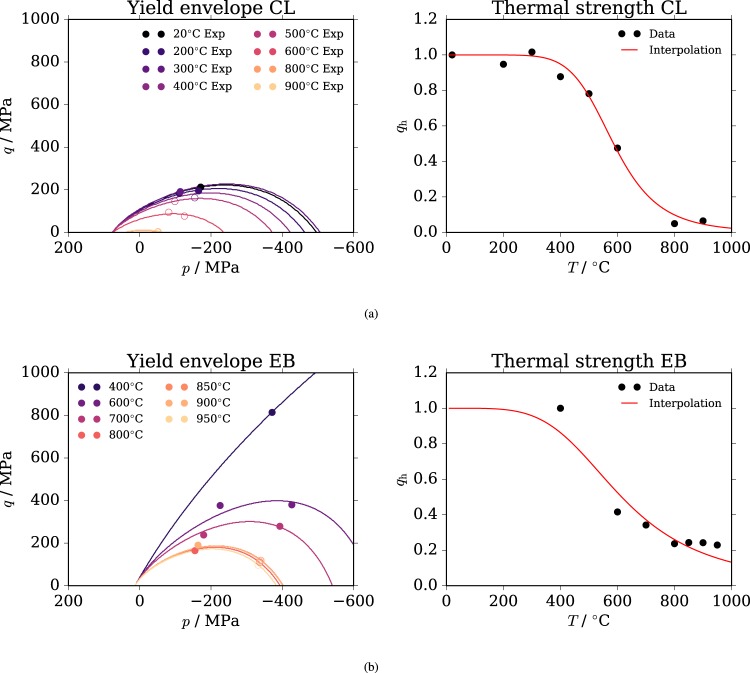
Calibration result. Calibration of the yield onset for Comiso Limestone (CL), on the top (**a**), and Escandorgue Basalt (EB), on the bottom (**b**). The dots represent the experimental values of plastic yield onset obtained from literature. The interested reader can consult the points’ location in the stress-strain curves in the supporting material provided with this manuscript (Fig. 4). The filled dots represent brittle deformation and the empty ones ductile deformation. The solid lines represent the envelope of yielding at the different temperatures and as a function of mean stress *p* for the two rocks. Every curve is obtained from the calibration of Eq. (2) with a different value of *q*_h_. Such values are then used to calibrate the thermal failure curve on the right images, which is in turn represented by Eq. (3).

In Fig. 1 we report the onset of inelasticity along with the plastic surface. It can be seen that those tests which exhibited ductile behaviour (open dots) are normally placed at higher pressure than the one corresponding to the maximum value of deviatoric strength in the yield surface. This means, assuming associated plastic-flow, that our model would predict negligible volumetric deformation for these ductile states with eventually a shift toward inelastic compaction for higher confinements. The sign of the inelastic volumetric deformation is indeed a good indicator of whether the deformation is brittle or ductile^42^. In the brittle regime the inelastic volumetric deformation is dilatant and in the ductile regime it is instead compactant, with isochoric deformation in the transition zone. This is confirmed by a parallel set of experiments performed on the same basalt^24^: at 100 MPa confinement there is increasingly small dilation up to roughly 800 °C, where the deformation becomes isochoric. At higher pressure and temperature a porosity reduction of 1–2% was observed in basalt, indicating plastic compaction^15^. Such irreversible compaction was confirmed in limestone, where permeability measurements indicated a non-reversible decrease with temperature^14^. This wide experimental evidence is in agreement with the proposed model, which exhibits exactly this transition in volumetric behaviour. We can conclude that taking the dilatancy value as a parameter for the BDT^42^ is not only convenient, but can be extended to low-porosity volcanic rocks at high temperatures as it fits the observations rather well and seems to be valid for both lithologies. In this framework, the model predicts that basalt is much more brittle and has greater strength at higher temperatures and confinements as compared to carbonate: the inelastic onset of carbonate at 20 °C is equivalent to the inelastic onset of basalt at 700 °C. Also, the transition is a function of both temperature and pressure.

**Figure 2:**
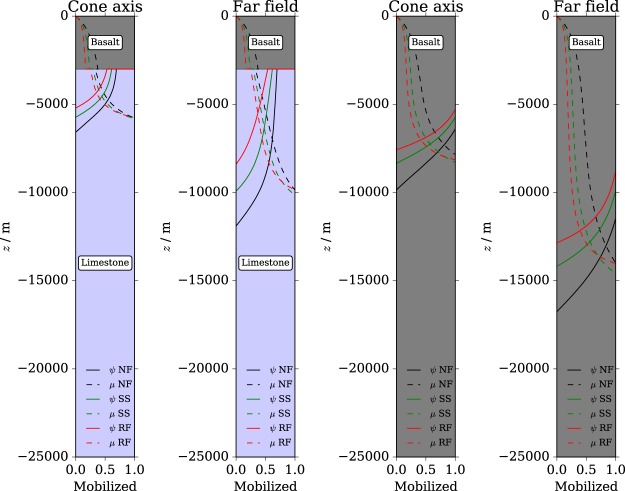
Strength profiles. Influence of the stress regime on the brittle-ductile transition at the volcanic cone axis and in the far field for the carbonatic basement and the basaltic one. The brittle-ductile transition is computed from the associated plastic flow in terms of a volumetric parameter *ψ*, that becomes negative for contractant volumetric plastic strain. Failure is indicated by the mobilized shear-strength *μ*, and occurs when *μ* ≥ 1. The stress regimes are indicated as Normal Faulting (NF), Strike-Slip (SS) and Reverse Faulting (RF).

As the majority of volcanoes lies within complex stress fields determined by the interplay of the mechanical properties of their basements, the regional tectonic forces and the intrusive processes in terms of dike emplacement, we have first investigated the influence of the stress regime on the depth at which the transition could take place. Results are shown in Fig. 2. In the case of a strato-volcano with a basaltic sequence resting upon a carbonatic basement, the depth of the BDT is appreciably smaller than in the case of a single basaltic lithology. Also, the transition depth is much lower below the volcanic cone, where the thermal gradient is much higher than in far field conditions. At the cone tip, the BDT is located at roughly 5–6 km depth in carbonates and at 8–10 km depth in basalts. Our results predict a BDT depth that is in agreement with the observed depth of attenuation of seismicity in strato-volcanoes with carbonatic basements such as Etna^43^ and Merapi^3,44^, and for purely basaltic basements such as Kilauea^45^, Mauna Loa^45^ and Piton de la Fournaise^46^. Furthermore, the BDT is located at roughly 9–13 km depth in carbonates and at 14–16 km depth in basalts when an average earth crust thermal gradient is applied (far field condition), in good agreement with previous models of the BDT^47^.

From the faulting regime analyses, we can observe that, based on the end term values that we have assumed for the deviatoric stress, the faulting regime does not play a major role in terms of strength mobilization *μ*, but has indeed an influence on the BDT depth given by the dilatant indicator sign(*ψ*). In all cases, reverse faulting (RF) seems to be the case for which the BDT is the shallowest, while in normal faulting (NF) conditions the BDT is found at a much higher depth. In strike slip faulting (SS) conditions, BDT depth is intermediate between reverse and normal faulting. The type of faulting regime becomes more influential with the volumetric inelastic deformation model at increasing depth. The influence of the faulting regime appears to be independent of both the lithology and the thermal gradient.

**Figure 3:**
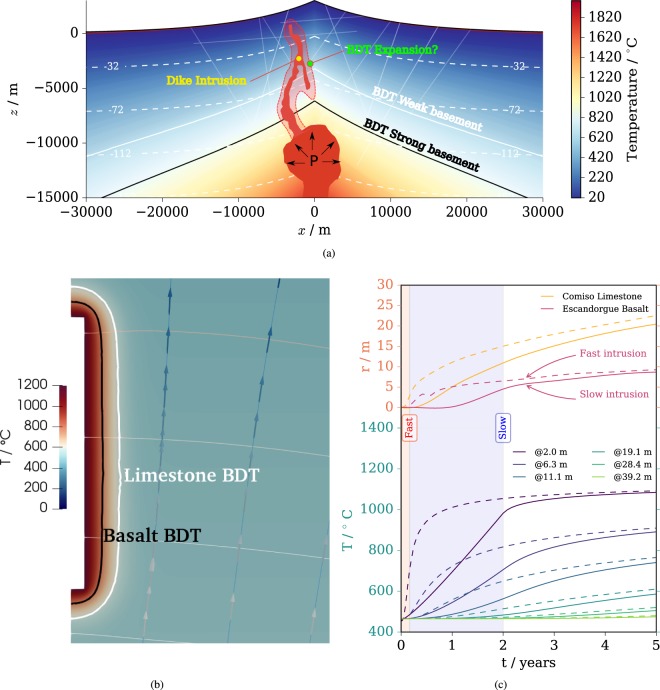
Volcanic model. Model illustrating the role of the rheology and lithology in a volcano globally, by defining the depth of the BDT line for the different lithological basements (**a**). The heat generates a perturbation in the hydraulic pressure and temperature around the dike (**b**). Evolution of the BDT position *r* during the dike process and for different intrusion speed and lithology along with the temperature *T* in the model at increasing distance from the dike (**c**).

We have built a schematic interpretation of the BDT in volcanic areas based on two-dimensional idealized volcanoes with carbonatic and purely basaltic basements, as shown in Fig. 3(a). The temperature distribution in the models is given along the location of the BDT line in the two cases and the iso-lines of pore pressure. In the case of carbonatic basement, the BDT is much shallower and can reach roughly 5 to 6 km below the cone tip. In basaltic settings, it is almost parallel to the isothermal line and reaches roughly 8–10 km below the cone tip. The fact that temperature might play a major role in defining the BDT depth is confirmed by seismic observations at the Krafla volcanic complex, where strong attenuation of seismicity has been observed at 2.7 km^48^. The temperature in the Krafla field at 2.0 km locally exceeds 900 °C, as confirmed by the extraction of rhyolitic magma from perforation cores^49^. As this contribution does not focus on a specific volcano, but rather on idealized typologies, the predicted depth of BDT could be influenced by other factors, such as, e.g., different lithologies involved or the impact that initial porosity has on rock strength, as was recently highlighted by several studies on volcanic and sedimentary rocks^50–52^. On the other hand, this contribution quantifies the non-negligible influence of lithology on the BDT depth by considering two typical end terms for active volcanoes, i.e. a microcristalline basaltic rock from Southern France representative of an extrusive environment typical of several effusive volcanoes and a cemented limestone representative of the thick sub-volcanic carbonate sedimentary sequences underlying several strato-volcano worldwide (e.g. Mt. Etna Volcano, Vesuvius, Campi Flegrei, Colli Albani complex, Merapi, Colima, Popocatepetl and Yellowstone among the others). Extensions to different lithologies and high-porosity rocks and more encompassing scenarios are foreseeable in the future.

We next analysed the consequences of magma intruding into the volcanic edifice (diking), which locally alters the state of the rock mass. Figure 3(b) depicts such alteration of the temperature and pore pressure fields after five years in the vicinity of a dike intruding the rock mass in 2 months. The highest temperature increase occurs around the dike (roughly 400 K increment at 10 m distance) as in this time frame the heat transport is mainly conductive and, because of the relatively low permeability of the rock, the advective component is low. On the other hand, temperature increase has the effect of decreasing water density, which generates a density driven flow directed upward, as indicated by the stream lines (perpendicular to the isobaric lines). The stress field is altered by the intrusion according to the following mechanisms: i) the over-pressure of 10 MPa applied by the dike; ii) the pore pressure increase that alters the effective stress and iii) the stress alteration caused by the thermally induced strain field in confined expansion conditions. The changes in the state of stress, along with the temperature field, have the consequence of altering the position of the BDT in the surroundings of the dike. The hotter rock closer to the dike will deform in a ductile manner while the cooler rocks far from the dike will fail in brittle mode.

The evolution in time of the BDT position *r* (distance from dike), along with the temperature *T* at different distances, has been computed for the different lithologies, considering a fast (2 months) and a slow (2 years) intrusion, both indicated in Fig. 3(c). The temperature evolution shows the effects of the fast vs. slow intrusion at increasing distance from the dike. At greater distances from the dike and at later time, the influence of the intrusion’s speed is lower, as the heating process is mainly conductive. The influence of the lithology in terms of the BDT location is evident, since the weaker carbonates exhibit ductile behaviour at lower temperature than the stronger basalts. Considering, for example, a fast intrusion, after a year the BDT will be moved by roughly 5 m for the basalts and more than 10 m for the carbonates. After five years, the difference between fast and slow intrusion is almost negligible, whereas the carbonate will still have a roughly double in size BDT distance compared to the one of the basalt. In this case, the BDT will be more than 20 m away from the dike. This might have strong implications in case of diffuse diking where each dike is spaced less than 50 m to the other. If that would be the case, almost all the rock mass between dikes would be in a ductile deformation mode. The shift in the BDT (which is essentially a rheological feature) and the local ductility induced, can partially explain the irregular patterns of seismicity before volcanic eruptions and the lack of a clear upward migration of foci during the magma transfer^1,7,37^. Close enough to the dike failure is ductile, hence likely a-seismic and plastic. Dilatant-brittle failure is therefore transferred further away from the dikes, which could explain the medium rarefaction^5–7^, a counter intuitive feature if one bears in mind the possible density increase associated to fracture filling caused by diking. Additionally, surface uplift could indeed be partially inhibited by the weak fracture zones forming^6,7^.

This interpretation invokes the rheological behaviour of the volcanic edifice as one of the primary controls of its response to magma pressurization and contributes to the understanding and interpreting of the role of the brittle-ductile transition in volcanic processes. The same approach could also be further employed to estimate the depth limit of seismicity occurrence in a volcanic area based on the BDT depth, or, alternatively, be used to estimate temperature profiles from seismic data. A relatively fresh topic of research concerns the possibility of extracting energy from volcanic areas, in so called super-hot or supercritical geothermal systems^53–55^. Our model could be propitiously employed not only to assess seismic risk (brittle or ductile failure mode), but also to perform preliminary numerical investigations on the possibility of enhancing permeability via stimulation and fracturing techniques.

## Methods

### Experimental data from literature

The rheological model is based on previously published mechanical data comprising triaxial compression experiments on limestone^14^ and basalt^15^. The tests were performed at different temperatures and confining pressures on specimens of Comiso Limestone (CL), a carbonatic formation that is found at 4–6 km depth below Mount Etna, Italy^14^. The specimens were retrieved from an outcrop at 50 km south of Mount Etna and solid bulk density of CL was reported to be 2468 kg m^−3^. Triaxial tests on dry samples were conducted at constant strain rate $\dot{\varepsilon }={10}^{-5}$ s^−1^, confining pressures of 50 and 100 MPa and temperatures ranging from 20 to 900 °C. The second material considered in this study is Escandorgue Basalt (EB), a glass-free basalt from Languedoc-Roussillon, France^15^. The authors of this work have reported the results of triaxial tests conducted at constant strain rate $\dot{\varepsilon }={10}^{-5}$ s^−1^, confining pressures of 100 and 300 MPa and temperatures ranging from 400 to 950 °C^15^. The initial bulk density of EB is roughly 2900 kg/m^3^. As expected, basalt is much more stable at high temperature and thermal degradation of strength is not initiated below 400 °C^15^.

### Model equations

The general framework of rate-independent plasticity is adopted and the plastic surface *f*_p_ is formulated in Biot’s effective stress space ***σ***′ = ***σ*** + *α*_b_*p*_w_**I**, with ***σ*** the total stress tensor, *p*_w_ the pore water pressure, *α*_b_ Biot’s coefficient (assumed to be equal to 1) and **I** the second-order identity tensor. The solid mechanical sign convention is applied throughout this study, i.e., tensile stresses and strains are positive. The Karush-Kuhn-Tucker loading-unloading conditions are defined as *f*_p_(***σ***′, *T*) ≤ 0, $\dot{\lambda }\ge 0$ and $\dot{\lambda }{f}_{{\rm{p}}}$(***σ***′, *T*) = 0, where *f*_p_(***σ***′, *T*) is the temperature and stress-dependent yield surface and $\dot{\lambda }$ is the plastic multiplier^56^. The plastic multiplier defines the magnitude of the rate of plastic strain ${\dot{{\boldsymbol{\varepsilon }}}}_{{\rm{p}}}=\dot{\lambda }\partial {g}_{{\rm{p}}}/\partial {\boldsymbol{\sigma }}^{\prime}$, which is normal to the plastic potential surface *g*_p_. If *g*_p_ = *f*_p_, as it is assumed in our work, the plastic potential is said to be associated and the plastic strain rate tensor is normal to the yield surface *f*_p_ (coaxial plasticity hypothesis is valid).

In this study, the yield surface is defined in the effective stress space via the mean effective stress and deviatoric stress invariants *p* and *q* defined as

1 $$\begin{array}{rcl}p & = & \frac{1}{3}{\rm{tr}}({\boldsymbol{\sigma }}^{\prime} )\\ q & = & {(\frac{3}{2}{\bf{s}}:{\bf{s}})}^{\frac{1}{2}},\end{array}$$

with the deviatoric effective stress tensor **s** = ***σ*** − tr(***σ***)**I**/3. The yield surface is inspired by research on the high-pressure behaviour of concrete^57^, here modified to account for thermal strength degradation, and writes

2 $${f}_{{\rm{p}}}(p,q,T)={[(1-{q}_{{\rm{h}}}(T)){(\frac{q}{3{\sigma }_{c}}+\frac{p}{{\sigma }_{c}})}^{2}+\frac{q}{{\sigma }_{c}}]}^{2}+{m}_{0}{q}_{{\rm{h}}}^{2}(T)(\frac{q}{3{\sigma }_{c}}+\frac{p}{{\sigma }_{c}})-{q}_{{\rm{h}}}^{2}(T),$$

where *σ*_c_ is the uniaxial compressive strength, *m*_0_ a frictional parameter and *q*_h_(*T*) is an internal functional dependent on temperature that defines the opening of the yield surface toward higher confinements, is bounded in the interval $[0,1]$ and has the following expression

3 $${q}_{{\rm{h}}}(T)=\frac{{q}_{{\rm{p}}}({{\boldsymbol{\varepsilon }}}_{{\rm{p}}})}{{[1+{(\alpha {\rm{\Delta }}T)}^{n}]}^{(1-\frac{1}{n})}}.$$

where *q*_p_(***ε***_p_) is a proper plastic hardening descriptor dependent on the plastic strain tensor ***ε***_p_. In the present case, *q*_p_ = 1 at the peak of stress (strength envelope) and *q*_p_ = *q*_p0_, with 0 ≤ *q*_p0_ ≤ 1, at the onset of inelasticity (yield envelope). The yield envelope corresponds to the points in the stress-strain triaxial curves in which the behaviour becomes inelastic.

**Figure 4:**
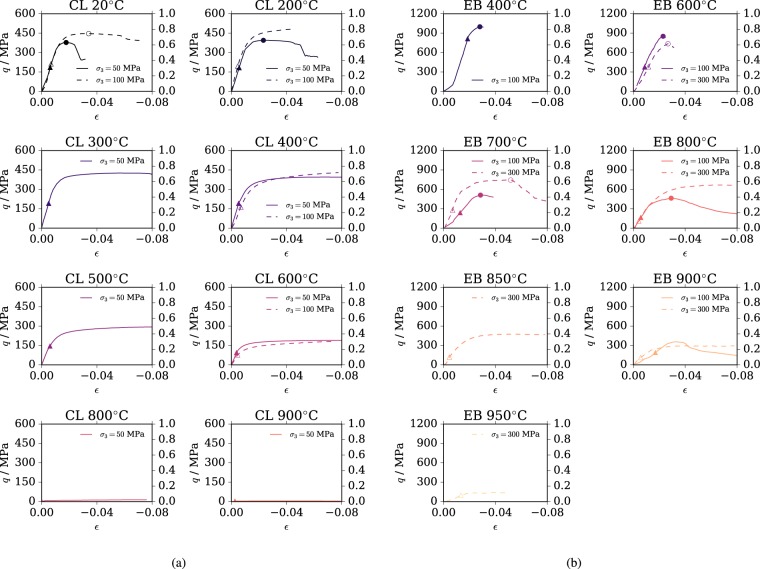
Experimental data. Deviatoric stress vs. vertical strain plots of triaxial tests results at different temperatures of Comiso Limestone (**a**) and Escandorgue Basalt (**b**). The curves were obtained from a digitalization of the plots reported in the original publications^14,15^. In the plots, we have indicated the points of deviation form linearity (plastic onset) with triangles and the points relative for the maximum strength in brittle conditions with circles.

Identifying the onset of inelasticity is a challenging procedure and experimentalists have often suggested porosity deviations or acoustic emissions’ onset as the marker indicating irreversible processes have started^42^. In the present work, given that such data are not available, we have followed an alternative procedure based on empirical considerations. More specifically, we have assumed that the pre-peak decrease of stiffness (deviation from linearity) constitutes the onset of inelastic strains. Because of the natural noise affecting the data, we have emplaced the following procedure: i) the stress-strain experimental points are approximated between zero and 3% of vertical deformation with a cubic spline using the function splprep available in Python’s scipy.interpolate package; ii) the stiffness is computed by taking the local derivative of the spline; iii) the onset of inelastic strain is taken as the pre-peak point at which the computed stiffness starts decreasing. The procedure gives consistent and satisfactory results, as one can visually confirm from Fig. 4. Concerning the final strength envelope, it is clear that it cannot be identified in ductile conditions, in which the material is continuously hardening and no peak is ever reached. In this case, only the yield stress is meaningful and the final failure condition is reached for very large values of plastic strain (cfr. Fig. 4).

In equation 3, Δ*T* = *T* − *T*_0_ is the difference between the current temperature *T* and the reference temperature *T*_0_, *α* and *n* are material parameters that define the shape of the thermal failure curve in the (Δ*T*, *q*_h_) space. Within the adopted framework, in brittle conditions the inelastic deformation is dilatant and in ductile conditions it is instead compactant, in agreement with previous studies^42^. Assuming the same convention, and assuming associated plasticity, the volumetric component of the inelastic deformation is ${\dot{\varepsilon }}_{{\rm{v}},{\rm{p}}}=\dot{\lambda }\partial {f}_{{\rm{p}}}/\partial p$.

### Calibration of material parameters

The five material parameters that describe the yield surface are *σ*_c_, *m*_0_, *q*_p0_, *α* and *n*. The values of *σ*_c_ and *m*_0_ can be estimated at fixed constant (reference) temperature Δ*T* = 0, which implies *q*_h_(*T*) = *q*_p_(***ε***_p_) and since both parameters are related to the final strength of the material, *q*_p_(***ε***_p_) = 1. The yield surface becomes

4 $${f}_{{\rm{p}}}(p,q)={(\frac{q}{{\sigma }_{{\rm{c}}}})}^{2}+{m}_{0}(\frac{q}{3{\sigma }_{{\rm{c}}}}+\frac{p}{{\sigma }_{{\rm{c}}}})-1,$$

which corresponds to the Menetrey-Williams failure surface^58^, which in turn is based on the Hoek-Brown failure surface^59^ written in the (*p*, *q*) stress space instead of the extremal principal stress space (*σ*_3_, *σ*_1_). Two points are necessary to obtain *σ*_c_ and *m*_0_. For the Comiso Limestone, we fitted the failure surface in equation 4 to pass through the maximum strength (peak stress) at 20 °C and at 50 and 100 MPa (see circles in Fig. 4) using the non-linear least squares curve_fit function, available in Python’s scipy.optimize, and obtained *σ*_c_ = 293.8 MPa and *m*_0_ = 3.857. Concerning Escandorgue Basalt, calibration is based on the maximum strength at 700 °C at confinements of 100 and 300 MPa, because tests at 400 °C were reported only with 100 MPa confinement, making them insufficient to calibrate a two-parametric curve. We obtain ${\sigma }_{{\rm{c}}}(T={700}^{\circ })=339.0$ MPa and *m*_0_ = 4.309 at 700 °C and, making the hypothesis of fixed values of σ_c_ with temperature, the curve fit at 400 °C with fixed σ_c_ = 339.0 MPa yields the final value of m_0_ = 26.11 for Escandorgue Basalt.

To calibrate *q*_p0_, which scales the yield surface at plastic onset and room temperature, the surface of equation 2 is fitted to pass through the plastic onset points (see triangles in Fig. 4) at 20 °C for CL and at 400 °C for EB, yielding *q*_p0_ = 0.5090 and *q*_p0_ = 0.8161, respectively. Repeating the process at the different temperatures yields a value of *q*_p0_(T*) for every temperature T*, so that *q*_p0_(T*)/*q*_p0_ indicates the values of *q*_h_(T*) at the different temperatures, controlling therefore the thermal degradation of the yield surface. Fitting the curve of equation 3 in the (*T*, *q*_h_(*T*)) space yields the values of the remaining parameters *α* and *n*, which are *α* = 0.001781 and *n* = 7.599 for CL and *α* = 0.001782 and *n* = 4.968 for EB. The final values of the parameters of the model are reported in Table 1.

**Table 1:** Model parameters for the rocks under consideration.

Parameter	Comiso Limestone	Escandorgue Basalt	Unit
*σ*_c_	293.8	339.0	MPa
*m*_0_	3.857	26.11	
*q*_p0_	0.5090	0.8161	
*α*	0.001781	0.001782	K^−1^
*n*	7.599	4.968	—

### Strength profile

In order to build a strength profile with depth, the state of stress in the rock mass needs to be computed. We made the hypothesis of lithostatic stress and hydrostatic pore pressure, so that at a given depth z

5 $$\begin{array}{rcl}{\sigma }_{{\rm{V}}}({\rm{z}}) & = & {\int }_{0}^{{\rm{z}}}\,{\rho }_{{\rm{s}}}({{\rm{z}}}^{\ast })g\,{{\rm{dz}}}^{\ast }\\ {p}_{{\rm{w}}}({\rm{z}}) & = & {\int }_{0}^{{\rm{z}}}\,{\rho }_{{\rm{w}}}({{\rm{z}}}^{\ast })g\,{{\rm{dz}}}^{\ast }+{p}_{0}\\ T({\rm{z}}) & = & {\int }_{0}^{{\rm{z}}}\,{{\rm{\Gamma }}}_{T}({{\rm{z}}}^{\ast })\,{{\rm{dz}}}^{\ast }+{T}_{0}\end{array}$$

with *ρ*_s_ and *ρ*_w_ the mass density of the rock and the water, respectively, and Γ_*T*_ is the thermal gradient. The maximum and minimum horizontal stresses are defined as *σ*_H_ = *k*_H_*σ*_V_ and *σ*_h_ = *k*_h_*σ*_V_, with *k*_H_ and *k*_h_ being multiplicative coefficients determining the stress regime. The stress regimes considered are Normal Faulting (NF), with ratios *σ*_H_/*σ*_V_ = 0.5 and *σ*_H_ = *σ*_h_, Strike-Slip (SS), with ratios *σ*_H_/*σ*_V_ = 1.3 and *σ*_h_/*σ*_V_ = 0.7 and Reverse Faulting (RF), with ratios *σ*_H_/*σ*_V_ = 1.5 and *σ*_H_ = *σ*_h_. The thermal gradient at the cone axis is assumed to be 100 K km^−1^ and in the far field 50 K km^−1^.

At a given depth z, temperature *T*, total ***σ*** and effective ***σ***′ stress tensors are known so that the acting mean effective ${p^{\prime} }_{{\rm{a}}}$ and deviatoric *q*_a_ stresses can also be computed; for a given pair $({p^{\prime} }_{{\rm{a}}},T)$, from equation 2 the available deviatoric strength $\bar{q}$ and the deviatoric to volumetric component ratio of plastic strain rate ${\dot{\varepsilon }}_{{\rm{v}},{\rm{p}}}/{\dot{\varepsilon }}_{{\rm{D}},{\rm{p}}}={[(\partial {f}_{{\rm{p}}}/\partial p)/(\partial {f}_{{\rm{p}}}/\partial q)]}_{{p}_{{\rm{a}}},\bar{q}}$ are computed. Finally, the mobilized strength $\mu ={q}_{{\rm{a}}}/\bar{q}$ and dilatancy coefficient $\psi =\arctan \,{[(\partial {f}_{{\rm{p}}}/\partial p)/(\partial {f}_{{\rm{p}}}/\partial q)]}_{{p}_{{\rm{a}}},\bar{q}}$ are calculated.

### Analysis of dike intrusion

The dike is assumed to be 20 m thick and 200 m long, it is intruding the volcano at a depth of 3 km, has a temperature of 1200 °C and exerts an excess isotropic total pressure of 10 MPa on the rock mass. These data are based on recent thermal propagation analyses of diking^60^. We investigate different scenarios, analyzing a rapid (2 months) and slow (2 years) dike intrusion. The initial conditions of pressure, temperature and mechanical stress are taken as equivalent to the global model. The mechanical response is elastic, the model is two-dimensional plain strain and the dike is assumed as’static’, i.e., non-propagating through the intact or previously fractured rock. The evolution of the BDT is finally calculated on the transient fields of temperature and effective stress resulting from the FE analysis. The system of partial differential equations (PDE) describing conservation of mass

6 $$(\varphi {\beta }_{{\rm{w}}}+\frac{1-\varphi }{{K}_{{\rm{s}}}})\frac{{\rm{d}}{p}_{{\rm{w}}}}{{\rm{d}}t}-3[\varphi {\alpha }_{{\rm{w}}}+{\alpha }_{{\rm{s}}}(\varphi -1)]\frac{{\rm{d}}T}{{\rm{d}}t}-\nabla \cdot [\frac{{\bf{k}}}{{\mu }_{{\rm{w}}}}(\nabla {p}_{{\rm{w}}}-{\rho }_{{\rm{w}}}{\bf{g}})]+\nabla \cdot \frac{{\rm{d}}{\bf{u}}}{{\rm{dt}}}={Q}_{{\rm{H}}},$$

energy

7 $$[\varphi \,{\rho }_{{\rm{w}}}\,{c}_{{\rm{w}}}+(1-\varphi ){\rho }_{{\rm{s}}}\,{c}_{{\rm{s}}}]\frac{{\rm{d}}T}{{\rm{dt}}}-\nabla \cdot [(\varphi {\lambda }_{{\rm{w}}}{\bf{I}}+(1-\varphi ){{\boldsymbol{\lambda }}}_{{\rm{s}}})\nabla T]-{\rho }_{{\rm{w}}}{c}_{{\rm{w}}}\frac{{\bf{k}}}{{\mu }_{{\rm{w}}}}(\nabla {p}_{{\rm{w}}}-{\rho }_{{\rm{w}}}{\bf{g}})\cdot \nabla T={Q}_{{\rm{T}}},$$

and momentum

8 $$\frac{E}{2(1-2\nu )\,(1+\nu )}\nabla (\nabla \cdot {\bf{u}}-3{\alpha }_{{\rm{s}}}{\rm{\Delta }}T)+\frac{E}{(1-2\nu )}{\nabla }^{2}{\bf{u}}-\nabla \cdot ({p}_{{\rm{w}}}{\bf{I}})+[\varphi {\rho }_{{\rm{w}}}+(1-\varphi ){\rho }_{{\rm{s}}}]{\bf{g}}={\bf{0}},$$

of the fluid-saturated rock is solved with open-source, object-oriented FE code OpenGeoSys (http://www.opengeosys.org/). The solution contains the time variation (transient) of the fields of unknown variables: pore pressure *p*_w_, temperature *T* and displacement **u**, describing the thermo-hydro-mechanical response of the rock. In the PDE system, for the rock, *ϕ* = 0.02 is the porosity, *ρ*_s_ = 2700 kg m^−3^ is the density, *E* = 40 GPa is Young’s modulus, *ν* = 0.25 is Poisson’s ratio, *K*_s_ is the bulk modulus of the solid phase, ${\bf{k}}=1\cdot {10}^{-15}\,{\bf{I}}$ m^2^ is the intrinsic permeability, ${\alpha }_{{\rm{s}}}=1\cdot {10}^{-5}$ is the linear thermal expansion coefficient, ***λ***_s_ = 3**I** W m^−1^ K^−1^ is the thermal conductivity and *c*_s_ = 950 J kg^−1^ K^−1^ is the specific heat capacity. Furthermore, **g** is the gravity acceleration vector and *Q*_H_ and *Q*_T_ are source terms. The fluid’s (water) bulk compressibility *β*_w_, linear thermal expansion *α*_w_, dynamic viscosity *μ*_w_, density *ρ*_w_, specific heat capacity *c*_w_ and thermal conductivity *λ*_w_ are state variables depending on pressure *p* and temperature *T* and are computed according to the IAPWS-97 standard for the thermodynamic properties of water and steam using the external library **freesteam** (http://freesteam.sourceforge.net/).

## Electronic supplementary material

Signed authorship update form

## Data Availability

The datasets generated during and/or analysed during the current study are available from the corresponding author. OpenGeoSys FE source code is available for download at http://www.opengeosys.org/.
