# Supplementary material for: The brittle-ductile transition in active volcanoes
Source: Sci Rep. 2019 Jan 15;9:143. doi: 10.1038/s41598-018-36505-x (PMC6333802; doi:10.1038/s41598-018-36505-x)
Supplement: Supplementary file 1 — Signed authorship update form [file 41598_2018_36505_MOESM1_ESM.pdf]

nature publishing group npg

In accordance to Nature Publishing Groups Authorship Policy we agree to change the authors of the manuscript as indicated below.

NAME OF JOURNAL: Scientific Reports

TITLE OF MANUSCRIPT: The brittle-ductile transition in active volcanoes

MANUSCRIPT NUMBER: SREP-18-21767A

CORRESPONDING AUTHORS NAME: Francesco Parisio

PREVIOUS AUTHOR NAMES:
Francesco Parisio, Thomas Nagel, Olaf Kolditz, Sergio Vinciguerra

UPDATED AUTHOR NAMES:
Francesco Parisio, Sergio Vinciguerra, Olaf Kolditz, Thomas Nagel

CHANGE TO AUTHOR LIST: We have re-evaluated individual contributions and modified the authors list in accordance.

Print Name	Signature	Date
Francesco Parisio	[illegible]	08/10/2018
Sergio Vinciguerra	Sergio Vinciguerra	06/10/2018
Olaf Kolditz	[illegible]	08/10/2018
Thomas Nagel	[illegible]	08/10/2018
